# A SuperLearner approach for predicting diabetic kidney disease upon the initial diagnosis of T2DM in hospital

**DOI:** 10.1186/s12911-025-02977-x

**Published:** 2025-03-26

**Authors:** Xiaomeng Lin, Chao Liu, Huaiyu Wang, Xiaohui Fan, Linfeng Li, Jiming Xu, Changlin Li, Yao Wang, Xudong Cai, Xin Peng

**Affiliations:** 1 Ningbo Institute of Chinese Medicine Research, Ningbo Municipal Hospital of Traditional Chinese Medicine (TCM), Affiliated Hospital of Zhejiang Chinese Medical University, No. 819, Liyuan North Road, Haishu District, Ningbo, 315010 China; 2 Yidu Cloud Technology Inc., Beijing, 100083 China; 3 Nanjing YiGenCloud Institute, Nanjing, 211899 China; 4 National Institute of Traditional Chinese Medicine Constitution and Preventive Treatment of Diseases, Beijing University of Chinese Medicine, Beijing, 100029 China; 5 Pharmaceutical Informatics Institute, College of Pharmaceutical Sciences, Zhejiang University, Hangzhou, 310058 China; 6 Department of Nephrology, Ningbo Municipal Hospital of Traditional Chinese Medicine (TCM), Affiliated Hospital of Zhejiang Chinese Medical University, Ningbo, 315010 China

**Keywords:** Type 2 diabetes, diabetic kidney disease, real-world data, machine learning, model interpretability, risk estimation

## Abstract

**Background:**

Diabetic kidney disease (DKD) is a serious complication of diabetes mellitus (DM), with patients typically remaining asymptomatic until reaching an advanced stage. We aimed to develop and validate a predictive model for DKD in patients with an initial diagnosis of type 2 diabetes mellitus (T2DM) using real-world data.

**Methods:**

We retrospectively examined data from 3,291 patients (1740 men, 1551 women) newly diagnosed with T2DM at Ningbo Municipal Hospital of Traditional Chinese Medicine (2011–2023). The dataset was randomly divided into training and validation cohorts. Forty-six readily available medical characteristics at initial diagnosis of T2DM from the electronic medical records were used to develop prediction models based on linear, non-linear, and SuperLearner approaches. Model performance was evaluated using the area under the curve (AUC). SHapley Additive exPlanation (SHAP) was used to interpret the best-performing models.

**Results:**

Among 3291 participants, 563 (17.1%) were diagnosed with DKD during median follow-up of 2.53 years. The SuperLearner model exhibited the highest AUC (0.7138, 95% confidence interval: [0.673, 0.7546]) for the holdout internal validation set in predicting any DKD stage. Top-ranked features were WBC_Cnt*, Neut_Cnt, Hct, and Hb. High WBC_Cnt, low Neut_Cnt, high Hct, and low Hb levels were associated with an increased risk of DKD.

**Conclusions:**

We developed and validated a DKD risk prediction model for patients with newly diagnosed T2DM. Using routinely available clinical measurements, the SuperLearner model could predict DKD during hospital visits. Prediction accuracy and SHAP-based model interpretability may help improve early detection, targeted interventions, and prognosis of patients with DM.

**Supplementary Information:**

The online version contains supplementary material available at 10.1186/s12911-025-02977-x.

## Background

Diabetic kidney disease (DKD) is a common microvascular complication of diabetes mellitus (DM) and is considered the leading cause of end-stage renal disease (ESRD) [1]. DKD is typically asymptomatic until reaching an advanced stage [2]. Although its progression can be slowed down through medication at an early stage, lifestyle changes, and careful blood sugar level management [3], its advanced stages are often irreversible and may result in ESRD, necessitating dialysis or kidney transplantation [4].

With approximately 30% of patients with DM developing DKD locally and globally [5–7], epidemiological data emphasise its significant prevalence. Furthermore, DKD has become the leading cause of dialysis [8], suggesting its severity. Moreover, a substantial portion of patients with abnormal clinical or laboratory measurements remain undiagnosed with DKD [9]. These findings suggest the need for a DKD early prediction model that can be used for risk communication.

Despite an increasing body of literature on DKD prediction models [10–12], including those specific to Asians or Chinese populations [13, 14], several factors limit the validity and clinical application of these models.

First, from the study design perspective, the selection of the study population in most previous studies was either unmatched with real-world hospital-visited patients with T2DM or introduced bias due to inappropriate requirements for data completeness. For instance, patients without baseline estimated glomerular filtration rate (eGFR) were excluded in certain studies [14–18], potentially introducing selection bias, as individuals who undergo creatinine testing due to physician suspicion of kidney disease are more likely to have pre-existing renal conditions [19, 20]. Consequently, the areas under the curve (AUC) of the models developed based on these cohorts may have been overestimated.

From a methodology perspective, the final best-performing models in previous studies were mostly classifiers of a single type, such as linear (e.g., Lasso) or non-linear (e.g., random forest) [11]. However, the complexity of potential predictors and their interactions suggests a need beyond a single algorithm. To date, no model has surpassed the predictive accuracy of any individual algorithm by appropriately weighting the contributions of each algorithm (e.g., SuperLearner). Moreover, several other factors hinder such investigations, including risk predictor selection based on univariate screening and the no mention of handling of missing data [21], revealing gaps in research methodology.

This study aims to address these gaps by applying and comparing the performance of multiple machine learning models for patients with T2DM. We utilized SuperLearner to combine predictions from various single algorithms for improving the overall prediction performance. Additionally, we used SHapley Additive exPlanations (SHAP) to improve the model interpretability by delineating how each feature contributes to the prediction outcome at the patient level.

## Methods

### Study design and population

This retrospective cohort study included patients initially diagnosed with T2DM at the Ningbo Municipal Hospital of Traditional Chinese Medicine between 2011 and 2023. All patients with at least one occurrence of T2DM were screened using ICD-9 or ICD-10 codes in the outpatient and inpatient departments. The baseline was defined as the time of earliest diagnosis of T2DM in each patient (also called ‘T0’ hereafter). A 6-month T0-centred interval was designated as the patient’s baseline time window.

The inclusion criteria were: (1) age ≥ 18 years; (2) at least one hospital visit (regardless of visit type) following the end of the baseline time window.

The exclusion criteria were: (1) diagnosis of DKD at any time before the end of the baseline time window, identified by a urinary albumin-to-creatinine ratio (UACR) ≥ 30 mg/g (urine microalbuminuria [mAlb] ≥ 30 mg, total protein [TP] − 24 h urine ≥ 180 mg), eGFR < 60 mL/min/1.73 m^2^ (the Chronic Kidney Disease Epidemiology Collaboration [CKD-EPI]) [22], or protein in the urine dipstick test ≥ 1 +; (2) pregnancy within the baseline time window; (3) presence of active infections within the baseline time window; (4) active cancer or malignancy within the baseline time window; (5) autoimmune disease within the baseline time window; (6) involvement of other renal diseases (e.g., urinary tract infection, polycystic kidney disease, glomerulonephritis) any time before the end of the baseline time window.

**Fig. 1:**
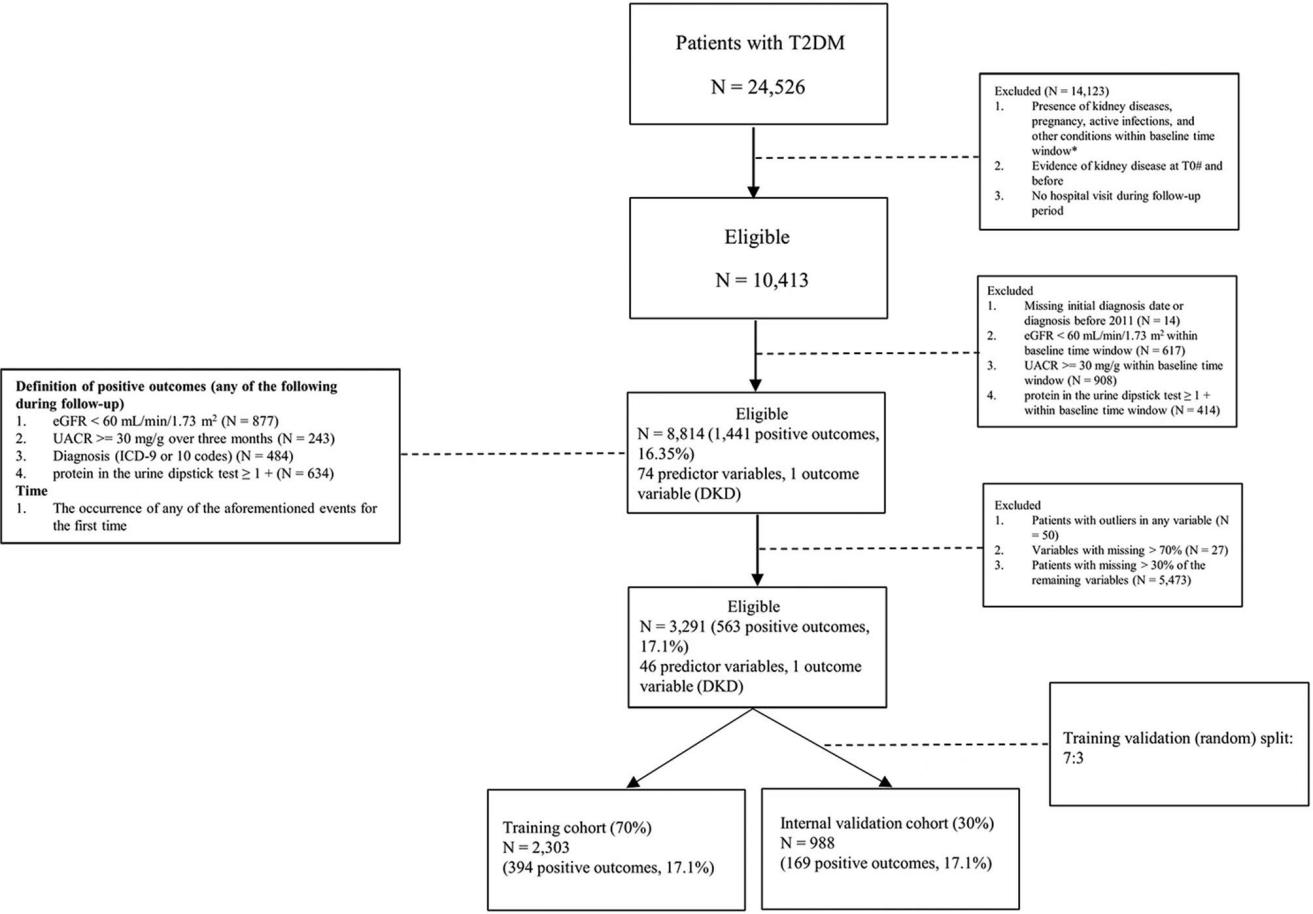
Diagram showing patient selection. *Baseline time window: a 6-month interval centred on the initial diagnosis of T2DM. #T0: initial diagnosis of T2DM. T2DM: type 2 diabetes mellitus; eGFR: estimated glomerular filtration rate; UACR: urinary albumin-to-creatinine ratio; DKD: diabetic kidney disease

Notably, we did not set thresholds for the required number of measurements for the key indices (e.g., eGFR) or the length of follow-up. Figure 1 shows the patient inclusion diagram.

### Candidate predictor variables and outcomes

The baseline data included 74 routinely measured characteristics extracted from the Medical Data Intelligence Platform related to patients’ initial T2DM diagnosis. Candidate predictor variables were classified into the following categories: (1) demographics (e.g., age at T0 and sex), (2) medical history (e.g., hypertension), (3) vital signs (e.g., systolic blood pressure), and (4) laboratory tests (e.g., routine blood tests, urinalysis, and biochemical analysis). All baseline predictor variables were collected within the patient’s baseline time window, and in case of multiple testing values for one parameter, the value closest to T0 was selected. A complete list of the predictor variables, attributes of each variable in the dataset, and their abbreviations in the analysis are displayed in Table S1.

The primary endpoint was the occurrence of DKD, regardless of the specific stage it reached, for the first time during follow-up. Specifically, the event was defined as any of the following: (1) persistent albuminuria (UACR ≥ 30 mg/g) (mAlb ≥ 30 mg, TP − 24 h Urine ≥ 180 mg) over 3 months; (2) eGFR < 60 mL/min/1.73 m^2^ [10]; (3) urinary protein in routine urine tests ≥ 1 +; (4) diagnosis of DKD according to ICD-9 or ICD-10 codes.

### Modelling

Patients with outliers in any predictor variables based on clinical experience were removed when preparing the data for modelling. Variables with more than 70% missing data were excluded. Subsequently, patients with missing data for more than 30% of the remaining variables were excluded. A comparison of variable distributions before and after applying missing-rate based filtering was conducted to ensure consistency. Missing data were verified to be missing at random [23]. Subsequently, missing values were addressed using the multivariate imputation by chained equations (MICE) method [24]. The variables used in the analyses were converted to numeric or binary values (e.g., 1 = male, 0 = female). The primary outcome variable was converted to either zero (negative, no DKD) or one (positive, DKD present).

The cohort dataset was randomly assigned to a training (70%, *N* = 2303) or validation (30%, *N* = 988) set before model development. Machine learning algorithms were used to develop prediction models. Typical linear models (i.e., multivariate logistic regression and Lasso [25]), typical non-linear models (i.e., random forest and extreme gradient boosting), and SuperLearner were used for model development [26]. Subsequently, the optimal parameters of the machine learning algorithms were obtained through cross-validation using the training set. Finally, the models’ performances were compared using the validation set, and the model with the highest AUC was selected as the final best-performing model.

SHapley Additive exPlanations (SHAP) was used to interpret the results of the best-performing prediction model by computing the contribution of each variable to the prediction [27]. SHAP values quantify each feature’s impact on a model’s prediction by assessing how the prediction changes when the feature is included or excluded across all possible combinations. To enhance interpretability, several SHAP visualization techniques were employed: the bee swarm plot ranked features by their overall importance across the population, the dependence plot illustrated SHAP values at the feature level across the study population, and the waterfall plot revealed SHAP contributions at the individual level.

### Model evaluation and validation

The performance of the best model (SuperLearner) was evaluated using AUC. The accuracy of the optimal cut-off value was assessed using sensitivity, specificity, and positive and negative predictive values (PPV and NPV, respectively).

The validation strategy, described in our previous study, was as follows: ‘By applying the final best-performing model to make predictions, patients in the holdout validation cohort were classified into two prognostic groups (i.e., high-risk group vs. low-risk group) based on their predicted probability of DKD and the selected cut-off probability. Their survival curves were compared using the Kaplan–Meier method’ [28].

### Statistical analysis

Unpaired two-tailed t-tests and Wilcoxon tests were used to compare the distributions of continuous variables. The median and quantile values were compared for variables that did not follow a normal distribution. The chi-square test was used to quantify the relationships between categorical variables (e.g., label balance between the training and validation sets). Missing values were imputed using the multivariate imputation by chained equations method (‘MICE’ R package). SuperLearner is implemented in the ‘SuperLearner’ R package. Statistical significance was set at *P* = 0.05, or *P* = 0.001 when adjusting for multiple comparisons as appropriate. Statistical analyses were performed using R version 4.0.1 (R Foundation for Statistical Computing, Vienna, Austria).

## Results

### Patient characteristics

Overall, 3,291 patients were included in the analysis (the initial diagnosis of T2DM was evenly distributed over the 12-year study period). The median age of the patients was 61 years (interquartile range [IQR], 50–69 years), and 1,551 (47.1%) of them were women. The median follow-up time (non-normal) was 2.53 years (IQR, 0.97–5.88 years) after the initial diagnosis of T2DM (i.e., T0). During the follow-up, 563 (17.1%) patients were diagnosed with DKD (Fig. 1).

### Predictor variables and outcomes

After filtering for missing rate-based data, we identified 3,291 patients and 47 variables (46 predictor variables and 1 outcome variable). A comparison of variable distributions before and after filtering confirmed that there were no significant differences in either the variables or the incidence rate. Baseline characteristics of patients who progressed to DKD and those who did not are presented in Table 1. RBC_Cnt, Hb, Hct, LDL, HDL, Ca, MCHC, Lymph_Cnt, TBIL, PLTHct, TC, ALT, Lymph_Per, and PLT_Cnt were significantly higher in patients without DKD than in those with DKD. A significant positive correlation was noted with age, urine pH, Mono_Per, PDW, DDimer, AST/ALT, RDWCV, creatinine, and MCV among those with DKD. No significant differences were found in other variables between the groups. All clinical variables were well-balanced between the training and validation sets (Table S2).

**Table 1:** Baseline demographic, clinical, and biological characteristics of patients who progressed to diabetic kidney disease and those who did not

Characteristics	DKD (*N* = 563)	Non-DKD (*N* = 2728)	*p*-value
**Demographics (** ***N*** ** = 2)**			
Age	66 (54–75)	60 (50–68)	< 0.001
Gender			0.413
Female	256	1295	
Male	307	1433	
**Lifestyle (** ***N*** ** = 2)**			
is_drinking	62 (11.01%)	360 (13.20%)	0.180
is_smoking	106 (18.83%)	518 (18.99%)	0.977
**Blood routine** (***N* = 21)**			
WBC_Cnt	6.1 (5.1–7.8)	6.3 (5.2–7.7)	0.495
Neut_Cnt	3.8 (2.9–5.1)	3.8 (2.9–5)	0.724
Hct	39.4 (36–42.6)	40.5 (37.5–43.9)	< 0.001
Hb	133 (121–145)	137.5 (127–150)	< 0.001
Lymph_Per	27.3 (20.9–33.55)	29.2 (22.6–35.6)	< 0.001
Baso_Cnt	0.02 (0–0.03)	0.02 (0.01–0.03)	< 0.001
RBC_Cnt	4.26 (3.89–4.71)	4.44 (4.10–4.85)	< 0.001
MCV	91.9 (88.9–95.1)	91.2 (88.48–93.8)	< 0.001
Lymph_Cnt	1.6 (1.3–2.1)	1.8 (1.4–2.2)	< 0.001
MCH	31.1 (29.8–32.3)	31 (30–32)	0.436
Neut_Per	62.8 (56–70.15)	61.7 (54.7–68.6)	0.049
Mono_Cnt	0.4 (0.3–0.5)	0.4 (0.3–0.5)	0.001
Mono_Per	6.8 (5.5–8.1)	6.1 (5.1–7.5)	< 0.001
PDW	16.5 (16.2–16.9)	16.4 (16.1–16.7)	< 0.001
Eos_Per	1.8 (0.9–3)	1.6 (0.9–2.7)	0.104
PLTHct	0.19 (0.15–0.22)	0.2 (0.16–0.23)	< 0.001
MCHC	338 (332.75–343.25)	340 (334–346)	< 0.001
PLT_Cnt	190 (154–230)	198.75 (163–239.6)	< 0.001
MPV	9.8 (9.1–10.75)	9.8 (9–10.7)	0.632
Baso_Per	0.4 (0.3–0.6)	0.4 (0.3–0.6)	0.454
RDWCV	13 (12.6–13.5)	12.8 (12.4–13.4)	< 0.001
**Biochemical analysis (** ***N*** ** = 13)**			
LDL	2.27 (1.75–2.76)	2.45 (1.91–3.01)	< 0.001
TC	4.34 (3.7–5.09)	4.58 (3.86–5.27)	< 0.001
Crea	61 (51–73)	59 (49.875–69)	< 0.001
HbA1c	7.6 (6.5–9.6)	7.4 (6.5–9)	0.118
HDL	1.13 (0.9–1.355)	1.185 (0.99–1.43)	< 0.001
TBIL	10.5 (7.65–14)	11.1 (8.3–15)	< 0.001
PA	236.9 (192.65–286.55)	242.8 (196.675–291.225)	0.248
TG	1.43 (1.055–2.06)	1.44 (1.02–2.1)	0.838
ALT	19 (13–29)	21 (14–32)	< 0.001
AST	19 (16–27)	20 (16–27)	0.966
ASTALT	1 (0.8–1.4)	0.9 (0.7–1.2)	< 0.001
Ca	2.23 (2.13–2.32)	2.25 (2.16–2.34)	< 0.001
Eos_Cnt	0.1 (0.06–0.2)	0.1 (0.05–0.17)	0.124
**Urinalysis (** ***N*** ** = 7)**			
pH	6.5 (6–7)	6 (5.5–6.5)	< 0.001
glu_qual	204 (36.23%)	1058 (38.78%)	0.278
ket_qual	47 (8.35%)	299 (10.96%)	0.078
uro_qual	6 (1.07%)	23 (0.843%)	0.790
nit_qual	15 (2.66%)	48 (1.76%)	0.209
ob_qual	69 (12.26%)	287 (10.52%)	0.257
bil_qual	3 (0.533%)	8 (0.293%)	0.620
**Blood coagulation function (** ***N*** ** = 1)**			
DDimer	133 (78–268.5)	105 (61.75–202)	< 0.001

Continuous variables are presented as medians (interquartile ranges), and categorical variables are presented as counts (percentages). The abbreviations of all analytical variables are detailed in Table S1

### Development, evaluation, and validation

After the missing-value imputation, we ensured the integrity of the dataset by thoroughly comparing the distribution of imputed values with the original observed data to verify consistency. Our analysis revealed no significant differences between the observed and imputed values across all 46 variables, and no significant outliers were identified in the feature set. Additionally, the incidence of DKD was consistent between the training and validation sets (17.1% vs. 17.1%, *P* > 0.99).

**Fig. 2:**
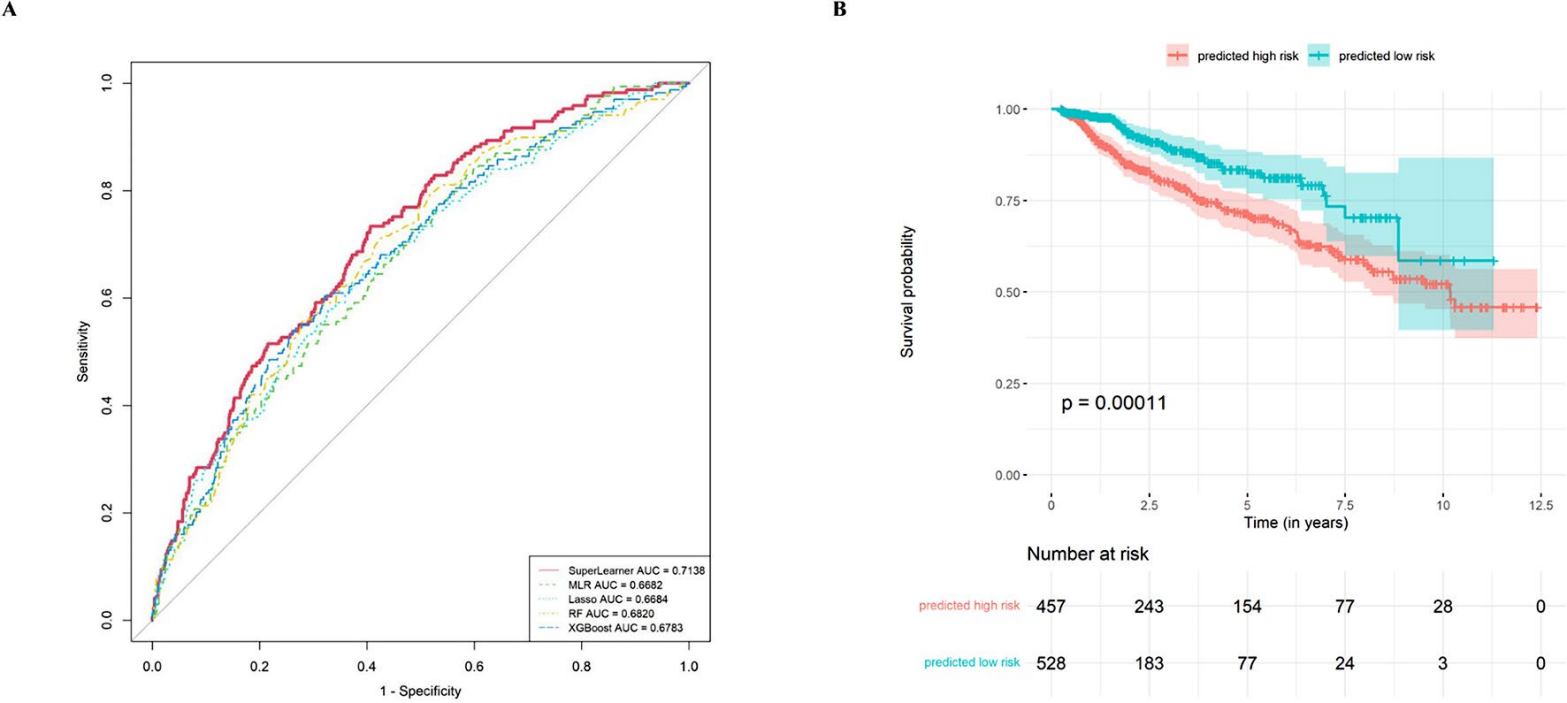
Models for predicting diabetic kidney disease and model evaluation of performance and validation. (**A**) Receiver operating characteristic curves for evaluating the discrimination ability of the model. SuperLearner had the highest area under the curve compared with the other models (*p* < 0.05*). *roc.test() was used for pairwise comparison of receiver operating characteristic curves and the results are presented in the Table S3. AUC: area under curve; MLR: multivariate logistic regression; RF: random forest. (**B**) Comparison of survival curves (end event: diabetic kidney disease) in different risk groups using SuperLearner (*P* < 0.01). *Regrettably, two individuals from the ‘predicted high-risk’ group and one individual from the ‘predicted low-risk’ group lacked the necessary time stamp for their final follow-up, necessitating their omission from the survival curve analysis

All variables (*N* = 46) were used as inputs for the five machine learning algorithms to predict the risk of DKD occurrence. The discrimination abilities were compared, and SuperLearner had the highest AUC (0.714, 95% confidence interval [CI] 0.673–0.755) (Fig. 2A, Table S3) and was thus chosen as the final best-performing model. In the validation cohort, the sensitivity, specificity, PPV, and NPV for differentiating DKD were 0.7337, 0.5910, 0.2702, and 0.9149, respectively (Table 2).

**Table 2:** Prediction accuracy of the superlearner estimating the risk of diabetic kidney disease in patients with type 2 diabetes mellitus and normal renal function

	Value (95% CI)	
Variable	Training cohort (*N* = 2303)	Validation cohort (*N* = 988)
#DKD	394 (17.11%)	169 (17.11%)
AUC	0.9378 (0.9272, 0.9484)	0.7138 (0.673, 0.7546)
Cutoff probability	0.15	0.15
Sensitivity, %	95.94 (93.49, 97.66)	73.37 (66.04, 79.87)
Specificity, %	67.73 (65.58, 69.83)	59.10 (55.64, 62.49)
PPV, %	38.03 (35.00, 41.13)	27.02 (23.00, 31.33)
NPV, %	98.78 (98.02, 99.30)	91.49 (88.78, 93.73)
Positive likelihood ratio	2.9732 (2.7775, 3.1827)	1.7938 (1.5869, 2.0277)
Negative likelihood ratio	0.0600 (0.0371, 0.0970)	0.4506 (0.3486, 0.5824)

*roc.test() for two receiver operator characteristic curves, *P* > 0.99

Abbreviations: AUC, area under the curve; CI, confidence interval; NPV, negative predictive value; PPV, positive predictive value

To further validate the model, patients in the validation cohort (*N* = 988) were categorised into two prognostic groups based on their DKD predicted probability by the best-performing model as follows: high-risk (459/988, predicted probability > 0.15) and low-risk (529/988, predicted probability ≤ 0.15) groups. Their survival curves were compared using the Kaplan–Meier method [29]. The difference between these groups was statistically significant (*P* < 0.01) (Fig. 2B).

### Explanation of risk factors

**Fig. 3:**
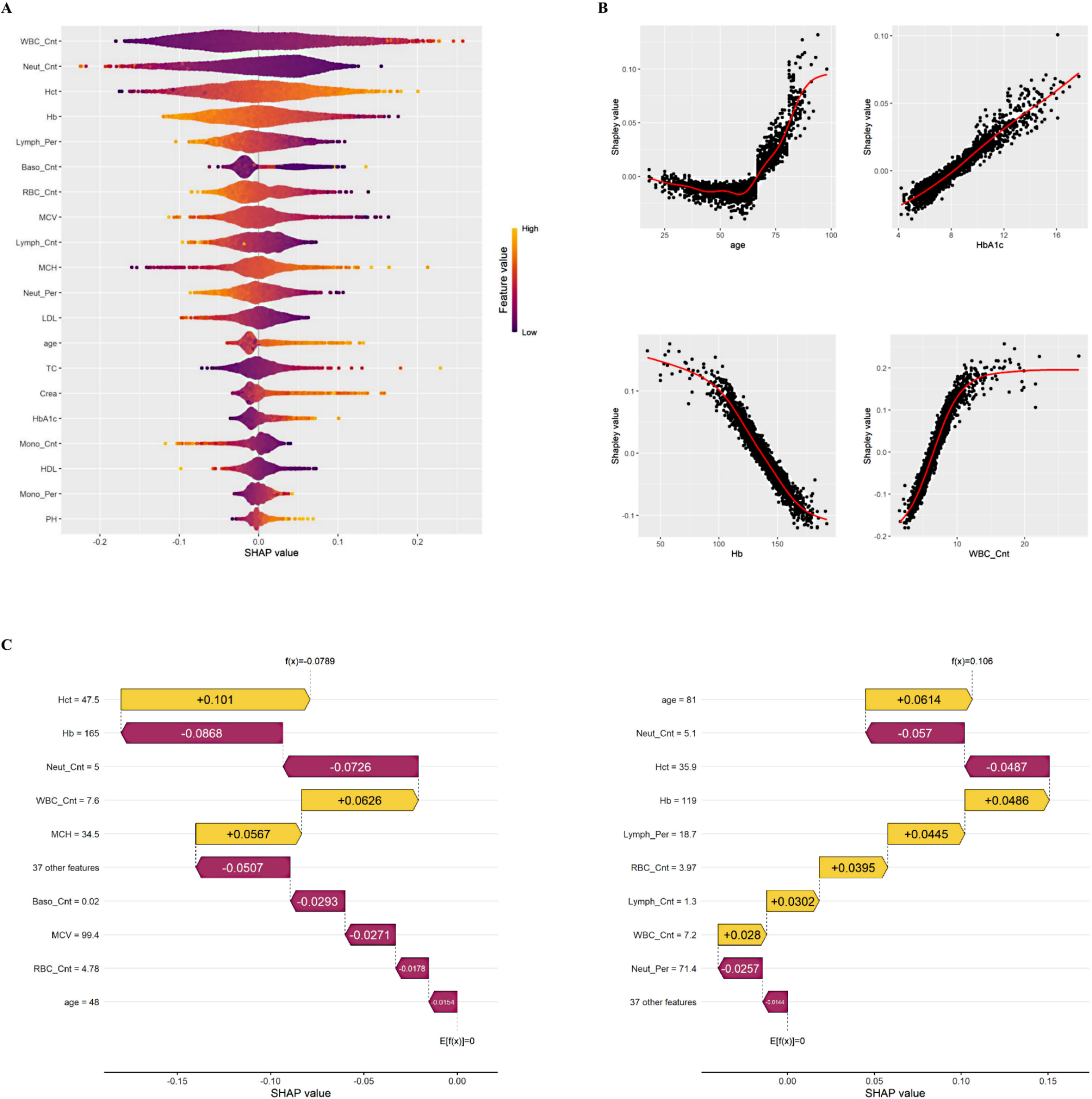
Model interpretability assessed using SHapley additive exPlanation. (**A**) The SuperLearner bee swarm plot depicts each variable’s importance for predicting diabetic kidney disease with type 2 diabetes mellitus and normal renal function (top 20). One dot per patient per feature is coloured according to an attribute value, where orange and purple represent higher and lower values, respectively. Features are sorted in decreasing order of importance, calculated as the average absolute SHAP value per feature. The abbreviations of all analytical variables are detailed in Table S1. (**B**) SHapley Additive explanation dependence plot of SuperLearner (selected four features), depicting how a single variable affects the prediction. SHapley Additive explanation values greater than zero for specific features suggested an increased risk of diabetic kidney disease. SHapley Additive explanation values below zero for specific features indicate a decreased risk of diabetic kidney disease. The remaining 16 from the top 20 plots are shown in Figure S1. (**C**) SHAP waterfall plot for patients with predicted low (left) and high (right) risk of developing DKD. SHAP value (left: -0.0789, right: 0.106). The base value at the bottom of the waterfall plot starts at zero. SHAP values shown inside yellow arrows correspond to input variables that ‘push’ the model towards predicting higher risk, whereas those in the magenta ‘push’ the model towards a lower predicted risk

SHAP was used to interpret the results of SuperLearner by computing the contribution of each variable to the prediction [13]. The SHAP summary plot (beeswarm) is shown in Fig. 3A. The importance plot ranked the variables contributing to DKD risk prediction from most to least important as patients’ baseline WBC_Cnt, Neut_Cnt, Hct, Hb, and so forth.

The SHAP dependence plots of the four selected variables included in SuperLearner are shown in Fig. 3B (the remaining top 20 plots are shown in Fig. S1). Higher SHAP values are associated with an increased risk of developing DKD. Older patients with higher WBC_Cnt, lower Neut_Cnt, higher Hct, and lower Hb levels had an increased risk of developing DKD. The feature value associated with zero SHAP can be used as a reference to determine the desired value of the variable, acting as a tipping point to distinguish between positive and negative contributions to DKD risk. A waterfall plot was used to illustrate how each patient’s specific clinical variables influenced their SHAP values, contributing to the overall prediction. Figure 3C provides examples: the left panel shows a 48-year-old male with a lower risk profile and a predicted probability of 0.1125 (< 0.15 threshold) for DKD, while the right panel shows an 81-year-old female with a higher predicted probability of 0.2721. Addressing modifiable risk factors, such as Hb, Lymph_Per, RBC_Cnt, Lymph_Cnt, and WBC_Cnt, may reduce this risk.

## Discussion

The present study included hospital-visiting patients initially diagnosed with T2DM without pre-existing renal diseases, using SuperLearner and SHAP to uncover complex relationships between predictors and DKD outcomes. In comparison to existing studies on developing prognostic models to predict DKD in patients with T2DM [10, 12], we identified new predictive markers (e.g., WBC_Cnt and urine pH) using innovative methods in representative populations. The study’s design, ensuring real-world relevance [30], improved clinical applicability, and the revealed associations between new markers and DKD offer new research avenues.

This study design enhanced the practicality and validity of the final model. First, we considered hospital visits in patients with T2DM; this population was neither general (e.g., the general healthy population) nor too specific (e.g., clinical research) [13]. Given the elevated DKD risk in T2DM patients, this approach emphasised the necessity for DKD screening [31, 32]. Patients initially diagnosed with T2DM typically undergo routine clinical tests, providing readily available measurements that make it easier to estimate their risk [16, 19, 20]. Second, using EMR-based automatic data extraction enhanced data-driven discovery, ranking previously uncommon markers like WBC_Cnt. This approach facilitated exploration of the association between DKD and blood dysfunction, such as anaemia, through the higher ranking of routine blood characteristics.

Unlike previous studies that solely employed a single classifier for risk modelling, this study conducted analyses using linear, non-linear (including ensemble learning algorithms), and SuperLearner models. The comparison of model performance revealed the superiority of SuperLearner over non-linear models, which, in turn, generally outperformed linear models in the validation set (Fig. 2A). Recognising the complexity of multiple interacting characteristics influencing the progression and risk of DKD [33, 34], this study provided data-driven evidence supporting clinical intuition and demonstrated these findings using data for the first time. To mitigate selection bias, we avoided excluding patients based on eGFR or other key indices (e.g., UACR) and opted for data-driven filtering. This increased the difficulty of improving the prediction model’s discriminative capability, measured using the AUC. This is because, in contrast to patients who are not required to be tested for creatinine, patients who are required to undergo creatinine testing usually have a higher likelihood of renal disease and suspicion of renal disease by physicians, making it easier to identify those with positive outcomes for the prediction model. Nevertheless, our study achieved a comparable AUC (0.7) compared to previous studies (0.6–0.8), thereby improving the practicality and validity of the final model [12].

For a more complex model, greater interpretability is required. One of the strengths of SHAP is its individualised explanation, which provides more explanation at a higher level of granularity [35]. In addition, the dependence plot is an accumulation of individualised explanations, which depicts the general association between variables and the risk of DKD and reveals a desired value for the variables. For instance, the risk of DKD escalated sharply at 65–67 years of age (Fig. 3B), consistent with findings from previous studies [13], presumably reflecting the nonlinear increase in the risk of kidney diseases and other age-associated conditions as individuals advance in age [36].

SuperLearner and SHAP confirmed the prognostic value of several common risk factors. The present study showed that age, glycated haemoglobin (HbA1c) levels, and serum creatinine levels were positively correlated with DKD risk (Fig. 3A, B). Previous studies have identified these markers as important variables for predicting long-term microvascular complications of diabetes [10, 13, 37, 38]. Specifically, older patients with T2DM and high HbA1c and creatinine levels had a higher risk of developing DKD. Moreover, the SHAP dependence plots provided tipping points where the DKD risk contribution of these variables switched from negative to positive; for example, 8 for HbA1c and 66 for age (Fig. 3B).

Infection, inflammation, and immunity are critical risk factors that affect DKD outcomes [39]. However, their role in early-stage DKD development has seldom been quantitatively investigated. In this study, WBC_Cnt and Neut_Cnt, reliable markers of systemic inflammation, infections, and immunity [40, 41], were ranked the highest among all potential predictors (Fig. 3A). We found that elevated WBC_Cnt and reduced Neut_Cnt were risk factors for DKD in patients newly diagnosed with T2DM and that these two characteristics at baseline were proportional to the risk of DKD even if they were in the clinically normal range. The identified association in this study and previous studies indicated that control of inflammatory activity and attention to immune status are critical to T2DM outcomes [42–44]. Patients with elevated WBC_Cnt and reduced Neut_Cnt at baseline should be closely monitored (e.g., more frequent routine screening for DKD).

In the present study, low Hb levels were associated with an increased risk of developing DKD. Although previous studies have demonstrated the predictive value of Hb for the risk of renal function decline in the early stages of DKD [45], the negative association between baseline Hb concentrations and the risk of follow-up DKD has often been overlooked [13, 46]. We speculate that patients with diabetes often have anaemia, which can lead to renal hypoxia and accelerate the progression of diabetic nephropathy [47, 48]. In contrast, other studies have shown that high Hb levels are also associated with an increased risk of diabetic nephropathy [49]. Maintaining an appropriate haemoglobin level is important for managing and preventing diabetic nephropathy.

Furthermore, higher baseline LDL levels were associated with a lower risk of progression to DKD. A previous study identified a similar association with marginal significance (*P* = 0.09) [13]. From a clinical perspective, elevated LDL levels are normally associated with an increased risk of DKD [50]. However, the current study showed the opposite association. We suspect this was due to the intervention provided. Because controlling lipid levels is significant, and lipids are one of the most intervenable indices [51, 52], patients with higher LDL-C levels are more aggressively managed with statins or other lipid-lowering therapies due to cardiovascular risk, which might confound the observed association. While statins lower LDL-C levels, they also have pleiotropic effects, such as anti-inflammatory properties and potential renal benefits, which could reduce the risk of DKD progression independently of LDL-C levels. Furthermore, patients on statins are more likely to receive comprehensive cardiovascular and renal risk management, potentially impacting DKD progression.

Notably, we identified new predictive markers, such as urine pH. Urine pH, which reflects the kidney’s ability to regulate the body’s acid-base balance and is related to diet, medication, and other factors [53], was positively associated with the risk of DKD. However, this finding has not been reported to date. Since urine pH detection is inexpensive and practical, it can serve as an auxiliary biomarker for DKD risk estimation. Additionally, this discovery provides valuable information for future research.

The findings from the present study have several clinical implications. First, based on our real-world study design and stringent inclusion and exclusion criteria, the model yielded a moderately good and realistic AUC, indicating good discriminatory ability. At the optimal threshold probability, the sensitivity and specificity were 0.7337 and 0.5910, respectively. Clinicians can adjust the threshold for better sensitivity (i.e., a lower risk threshold) or specificity (i.e., a higher risk threshold) case-by-case. Second, the dependence plots generated by SHAP provide index-wise cut-off points for DKD risk alteration, which, when combined with mechanism validation, can be used to facilitate clinical decision-making, such as targeted interventions. Third, at the individual patient level, SHAP waterfall plots allow clinicians to pinpoint the modifiable risk factors contributing most to a patient’s predicted risk. This personalized insight helps guide interventions such as lifestyle changes, medication adjustments, or further diagnostics, ultimately reducing risk and improving outcomes for that specific patient.

This study has some limitations as well. First, the analysis was based on patient data from a single centre, which may limit the generalisability of these findings to other populations, including those from different regions or ethnic backgrounds. The data also reflected centre-specific characteristics, including the lack of established risk factors, such as diabetic retinopathy. Multicentre studies and external validations are required to confirm these results. Second, a prospective study is required to confirm the usefulness of the developed model. The third limitation of our study is the relatively short follow-up period, which may limit the ability to identify later-onset outcomes and constrain the estimation of risk within the observed duration. Future studies with extended follow-up durations would help identify these cases and provide a more accurate assessment of the long-term effects, while adjustments for known risk factors would further enhance the validity and clinical applicability of the results. Nonetheless, the study design and large dataset generated by our Medical Data Intelligence Platform, the data-driven analytical approach, and the interpretability greatly improved the validity and robustness of the final model.

## Conclusions

In conclusion, we used real-world data and novel analytical methods and developed a SHAP-based explainable SuperLearner model to predict DKD in patients with T2DM with hospital visits. The resulting model revealed discoveries via risk estimation (e.g., markers), which can improve patient outcomes through easy-to-conduct early detection and more targeted interventions.

## Electronic Supplementary Material

Below is the link to the electronic supplementary material.

Supplementary Material 1

Supplementary Material 2

## Data Availability

The datasets used and/or analysed during the current study are available from the corresponding author on reasonable request.

## Abbreviations

WBC_Cnt: White blood cell count
Neut_Cnt: Neutrophil Counts
Hct: Hematocrit
Hb: Hemoglobin
Lymph_Per: Lymphocyte percentage
Baso_Cnt: Basophil counts
RBC_Cnt: Red blood cell count
MCV: Mean corpuscular volume
Lymph_Cnt: Lymphocyte count
MCH: Mean Corpuscular Hemoglobin
Neut_Per: Neutrophil percentage
LDL: Low density lipoprotein
Age: Age
TC: Total cholesterol
Crea: Creatinine
HbA1c: Haemoglobin A1c
Mono_Cnt: Monocyte count
HDL: High density lipoprotein
Mono_Per: Monocyte percentage
PH: Urine potential of hydrogen
PDW: Platelet Distribution Width
Eos_Per: Eosinophil percentage
TBIL: Total bilirubin
PLTHct: Plateletcrit
PA: Prealbumin
TG: Triglyceride
is_smoking: Smoking
MCHC: Mean corpuscular hemoglobin concentration
ALT: Alanine transaminase
PLT_Cnt: Platelet count
AST: Aspartate aminotransferase
DDimer: D-Dimer
glu_qual: Glucose qualitative
MPV: Mean platelet volume
Baso_Per: Basophil percentage
RDW-CV (RDWCV): Red cell volume distribution width -coefficient of variation
is_drinking: Drinking
ket_qual: Ketone qualitative
AST/ALT (ASTALT): Aspartate aminotransferase/Alanine transaminase
Ca: Calcium
Eos_Cnt: Eosinophil count
uro_qual: Urobilinogen qualitative
nit_qual: Nitrite qualitative
gender: Gender
ob_qual: Occult blood qualitative
bil_qual: Bilirubin qualitative

## References

[1] Tuttle KR, Bakris GL, Bilous RW, Chiang JL, De Boer IH, Goldstein-Fuchs J, et al. Diabetic kidney disease: a report from an ADA Consensus Conference. Diabetes Care. 2014;37(10):2864–83. 25249672 10.2337/dc14-1296 PMC4170131

[2] Mu X, Wu A, Hu H, Zhou H, Yang M. Prediction of diabetic kidney disease in newly diagnosed type 2 diabetes mellitus. Diabetes, Metabolic Syndrome and Obesity. 2023:2061–75. 10.2147/DMSO.S417300 PMC10337686 37448880

[3] McGrath K, Edi R. Diabetic kidney disease: diagnosis, treatment, and prevention. Am Family Phys. 2019;99(12):751–9. 31194487

[4] Braun MM, Khayat M. Kidney Disease: End-Stage Renal Disease. FP essentials. 2021;509:26–32. 34643362

[5] Gheith O, Farouk N, Nampoory N, Halim MA, Al-Otaibi T. Diabetic kidney disease: world wide difference of prevalence and risk factors. J nephropharmacology. 2016;5(1):49. PMC5297507 28197499

[6] Society CD, Association CM. A nationwide retrospective analysis on chronic diabetic complications and related macrovascular diseases of in-patients with diabetes during 1991–2000. Zhongguo yi xue ke xue yuan xue bao. Acta Academiae Medicinae Sinicae. 2002;24(5):447–51. 12905762

[7] Pelle MC, Provenzano M, Busutti M, Porcu CV, Zaffina I, Stanga L, et al. Up-date on diabetic nephropathy. Life. 2022;12(8):1202. 36013381 10.3390/life12081202 PMC9409996

[8] Bouhairie VE, McGill JB. Diabetic kidney disease. Mo Med. 2016;113(5):390. 30228506 PMC6139827

[9] Wei J, Wang B, Shen F-j, Zhang T-t, Duan Z, Zhou D. -m. Diagnostic value of triglyceride and cystatin C ratio in diabetic kidney disease: a retrospective and prospective cohort study based on renal biopsy. BMC Nephrol. 2022;23(1):1–8. 35896961 10.1186/s12882-022-02888-3 PMC9327235

[10] Jiang W, Wang J, Shen X, Lu W, Wang Y, Li W, et al. Establishment and validation of a risk prediction model for early diabetic kidney disease based on a systematic review and meta-analysis of 20 cohorts. Diabetes Care. 2020;43(4):925–33. 32198286 10.2337/dc19-1897

[11] González-Rocha A, Colli VA, Denova-Gutiérrez E. Peer Reviewed: Risk Prediction Score for Chronic Kidney Disease in Healthy Adults and Adults With Type 2 Diabetes: Systematic Review. Prev Chronic Dis. 2023;20. 10.5888/pcd20.220380 PMC10159345 37079751

[12] Slieker RC, van der Heijden AA, Siddiqui MK, Langendoen-Gort M, Nijpels G, Herings R et al. Performance of prediction models for nephropathy in people with type 2 diabetes: systematic review and external validation study. BMJ. 2021;374. 10.1136/bmj.n2134 PMC8477272 34583929

[13] Dong Z, Wang Q, Ke Y, Zhang W, Hong Q, Liu C, et al. Prediction of 3-year risk of diabetic kidney disease using machine learning based on electronic medical records. J Translational Med. 2022;20(1):1–10. 10.1186/s12967-022-03339-1 PMC8959559 35346252

[14] Low S, Lim SC, Zhang X, Zhou S, Yeoh LY, Liu YL, et al. Development and validation of a predictive model for chronic kidney disease progression in type 2 diabetes mellitus based on a 13-year study in Singapore. Diabetes Res Clin Pract. 2017;123:49–54. 27923172 10.1016/j.diabres.2016.11.008

[15] Lin C-C, Niu MJ, Li C-I, Liu C-S, Lin C-H, Yang S-Y, et al. Development and validation of a risk prediction model for chronic kidney disease among individuals with type 2 diabetes. Sci Rep. 2022;12(1):4794. 35314714 10.1038/s41598-022-08284-z PMC8938464

[16] Allen A, Iqbal Z, Green-Saxena A, Hurtado M, Hoffman J, Mao Q, et al. Prediction of diabetic kidney disease with machine learning algorithms, upon the initial diagnosis of type 2 diabetes mellitus. BMJ Open Diabetes Res Care. 2022;10(1):e002560. 35046014 10.1136/bmjdrc-2021-002560 PMC8772425

[17] Ou S-M, Tsai M-T, Lee K-H, Tseng W-C, Yang C-Y, Chen T-H, et al. Prediction of the risk of developing end-stage renal diseases in newly diagnosed type 2 diabetes mellitus using artificial intelligence algorithms. BioData Min. 2023;16(1):8. 36899426 10.1186/s13040-023-00324-2 PMC10007785

[18] Hui M, Ma J, Yang H, Gao B, Wang F, Wang J, et al. ESKD Risk Prediction Model in a Multicenter Chronic Kidney Disease Cohort in China: A Derivation, Validation, and Comparison Study. J Clin Med. 2023;12(4):1504. 36836039 10.3390/jcm12041504 PMC9965616

[19] ElSayed NA, Aleppo G, Aroda VR, Bannuru RR, Brown FM, Bruemmer D, et al. 11. Chronic Kidney Disease and Risk Management: Standards of Care in Diabetes—2023. Diabetes Care. 2023;46(Supplement1):S191–202. 36507634 10.2337/dc23-S011 PMC9810467

[20] Liew A, Bavanandan S, Prasad N, Wong MG, Chang JM, Eiam-Ong S, et al. Asian Pacific Society of Nephrology clinical practice guideline on diabetic kidney disease–an executive summary. Nephrology. 2020;25(11):809–17. 33111435 10.1111/nep.13804

[21] Collins GS, Omar O, Shanyinde M, Yu L-M. A systematic review finds prediction models for chronic kidney disease were poorly reported and often developed using inappropriate methods. J Clin Epidemiol. 2013;66(3):268–77. 23116690 10.1016/j.jclinepi.2012.06.020

[22] Levey AS, Stevens LA, Schmid CH, Zhang Y, Castro AF III, Feldman HI, et al. A new equation to estimate glomerular filtration rate. Ann Intern Med. 2009;150(9):604–12. 10.7326/0003-4819-150-9-200905050-00006 PMC2763564 19414839

[23] Rouzinov S, Berchtold A. Regression-based approach to test missing data mechanisms. Data. 2022;7(2):16.

[24] Van Buuren S, Groothuis-Oudshoorn K. mice: Multivariate imputation by chained equations in R. J Stat Softw. 2011;45:1–67.

[25] Tibshirani R. Regression shrinkage and selection via the lasso. J Roy Stat Soc: Ser B (Methodol). 1996;58(1):267–88.

[26] Van der Laan MJ, Polley EC, Hubbard AE. Super learner. Stat Appl Genet Mol Biol. 2007;6(1). 10.2202/1544-6115.1309 17910531

[27] Lundberg SM, Lee S-I. A unified approach to interpreting model predictions. Adv Neural Inf Process Syst. 2017;30.

[28] Wang P, Liu C, Wei Z, Jiang W, Sun H, Wang Y et al. Nomogram for Predicting Early Mortality after Umbilical Cord Blood Transplantation in Children with Inborn Errors of Immunity. J Clin Immunol. 2023:1–14. 10.1007/s10875-023-01505-8 PMC10354135 37155023

[29] Jager KJ, Van Dijk PC, Zoccali C, Dekker FW. The analysis of survival data: the Kaplan–Meier method. Kidney Int. 2008;74(5):560–5. 18596735 10.1038/ki.2008.217

[30] Spanopoulos D, Okhai H, Zaccardi F, Tebboth A, Barrett B, Busse M, et al. Temporal variation of renal function in people with type 2 diabetes mellitus: a retrospective UK clinical practice research datalink cohort study. Diabetes Obes Metabolism. 2019;21(8):1817–23. 10.1111/dom.13734 PMC6767485 30941882

[31] Hussain S, Jamali MC, Habib A, Hussain MS, Akhtar M, Najmi AK. Diabetic kidney disease: An overview of prevalence, risk factors, and biomarkers. Clin Epidemiol Global Health. 2021;9:2–6.

[32] Thomas MC, Brownlee M, Susztak K, Sharma K, Jandeleit-Dahm KA, Zoungas S, et al. Diabetic kidney disease. Nat reviews Disease primers. 2015;1(1):1–20. 10.1038/nrdp.2015.18 PMC7724636 27188921

[33] Shaw J, Rudzicz F, Jamieson T, Goldfarb A. Artificial intelligence and the implementation challenge. J Med Internet Res. 2019;21(7):e13659. 31293245 10.2196/13659 PMC6652121

[34] Clark RR, Hou J. Three machine learning algorithms and their utility in exploring risk factors associated with primary cesarean section in low-risk women: A methods paper. Res Nurs Health. 2021;44(3):559–70. 33651381 10.1002/nur.22122 PMC8068617

[35] Wang D, Thunéll S, Lindberg U, Jiang L, Trygg J, Tysklind M. Towards better process management in wastewater treatment plants: Process analytics based on SHAP values for tree-based machine learning methods. J Environ Manage. 2022;301:113941. 34731954 10.1016/j.jenvman.2021.113941

[36] Shen X, Wang C, Zhou X, Zhou W, Hornburg D, Wu S et al. Nonlinear dynamics of multi-omics profiles during human aging. Nat Aging 2024:1–16. 10.1038/s43587-024-00692-2 PMC11564093 39143318

[37] Saputro SA, Pattanaprateep O, Pattanateepapon A, Karmacharya S, Thakkinstian A. Prognostic models of diabetic microvascular complications: a systematic review and meta-analysis. Syst reviews. 2021;10(1):1–11. 10.1186/s13643-021-01841-z PMC8561867 34724973

[38] Khanam PA, Hoque S, Begum T, Habib SH, Latif ZA. Microvascular complications and their associated risk factors in type 2 diabetes mellitus. Diabetes Metabolic Syndrome: Clin Res Reviews. 2017;11:S577–81. 10.1016/j.dsx.2017.04.007 28455164

[39] Rayego-Mateos S, Rodrigues-Diez R, Fernandez-Fernandez B, Mora-Fernández C, Marchant V, Donate-Correa J et al. Targeting inflammation to treat diabetic kidney disease: The road to 2030. Kidney Int. 2022. 10.1016/j.kint.2022.10.030 36470394

[40] Winter L, Wong LA, Jerums G, Seah J-m, Clarke M, Tan SM, et al. Use of readily accessible inflammatory markers to predict diabetic kidney disease. Front Endocrinol. 2018;9:225. 10.3389/fendo.2018.00225 PMC5992400 29910771

[41] Ryu S, Shin JW, Kwon S, Lee J, Kim YC, Bae Y-S et al. Siglec-F–expressing neutrophils are essential for creating a profibrotic microenvironment in renal fibrosis. J Clin Investig. 2022;132(12). 10.1172/JCI156876 PMC9197522 35482420

[42] Guo W, Song Y, Sun Y, Du H, Cai Y, You Q, et al. Systemic immune-inflammation index is associated with diabetic kidney disease in Type 2 diabetes mellitus patients: Evidence from NHANES 2011–2018. Front Endocrinol. 2022;13:1071465. 10.3389/fendo.2022.1071465 PMC9763451 36561561

[43] Liu L, Gao B, Wang J, Yang C, Wu S, Wu Y, et al. Clinical significance of single and persistent elevation of serum high-sensitivity C-reactive protein levels for prediction of kidney outcomes in patients with impaired fasting glucose or diabetes mellitus. J Nephrol. 2021;34:1179–88. 32880885 10.1007/s40620-020-00848-4

[44] Schei J, Stefansson VTN, Eriksen BO, Jenssen TG, Solbu MD, Wilsgaard T, et al. Association of TNF receptor 2 and CRP with GFR decline in the general nondiabetic population. Clin J Am Soc Nephrology: CJASN. 2017;12(4):624. 10.2215/CJN.09280916 PMC5383389 28153935

[45] Yamanouchi M, Furuichi K, Shimizu M, Toyama T, Yamamura Y, Oshima M, et al. Serum hemoglobin concentration and risk of renal function decline in early stages of diabetic kidney disease: a nationwide, biopsy-based cohort study. Nephrol Dialysis Transplantation. 2022;37(3):489–97. 10.1093/ndt/gfab185 34028524

[46] Keane WF, Zhang Z, Lyle PA, Cooper ME, de Zeeuw D, Grunfeld J-P, et al. Risk scores for predicting outcomes in patients with type 2 diabetes and nephropathy: the RENAAL study. Clin J Am Soc Nephrol. 2006;1(4):761–7. 17699284 10.2215/CJN.01381005

[47] Sahay M, Kalra S, Badani R, Bantwal G, Bhoraskar A, Das A et al. Diabetes and anemia: International diabetes federation (IDF)–Southeast Asian Region (SEAR) position statement. Diabetes & Metabolic Syndrome: Clinical Research & Reviews. 2017;11:S685–S95. 10.1016/j.dsx.2017.04.026 28483426

[48] Liu J, Wei Q, Guo C, Dong G, Liu Y, Tang C, et al. Hypoxia, HIF, and associated signaling networks in chronic kidney disease. Int J Mol Sci. 2017;18(5):950. 28468297 10.3390/ijms18050950 PMC5454863

[49] Xin S, Zhao X, Ding J, Zhang X. Association between hemoglobin glycation index and diabetic kidney disease in type 2 diabetes mellitus in China: A cross-sectional inpatient study. Front Endocrinol. 2023;14:1108061. 10.3389/fendo.2023.1108061 PMC10031087 36967789

[50] Siddiqui K, George TP, Nawaz SS, Yaslam M, Almogbel E, Al-Rubeaan K. Significance of glycated LDL in different stages of diabetic nephropathy. Diabetes Metabolic Syndrome: Clin Res Reviews. 2019;13(1):548–52. 10.1016/j.dsx.2018.11.023 30641763

[51] Dake AW, Sora ND. Diabetic dyslipidemia review: an update on current concepts and management guidelines of diabetic dyslipidemia. Am J Med Sci. 2016;351(4):361–5. 27079341 10.1016/j.amjms.2016.01.020

[52] Jialal I. Management of diabetic dyslipidemia: Navigating the new American and European Guidelines. Diabetes Metabolic Syndrome. 2020;14(5):877–9. 32562865 10.1016/j.dsx.2020.06.010

[53] Miki A, Hashimoto Y, Tanaka M, Kobayashi Y, Wada S, Kuwahata M, et al. Urinary pH reflects dietary acid load in patients with type 2 diabetes. J Clin Biochem Nutr. 2017;61(1):74–7. 28751813 10.3164/jcbn.16-118 PMC5525012

