# Supplementary figures and images for: A SuperLearner approach for predicting diabetic kidney disease upon the initial diagnosis of T2DM in hospital
Source: BMC Med Inform Decis Mak. 2025 Mar 26;25:148. doi: 10.1186/s12911-025-02977-x (PMC11948915; doi:10.1186/s12911-025-02977-x)

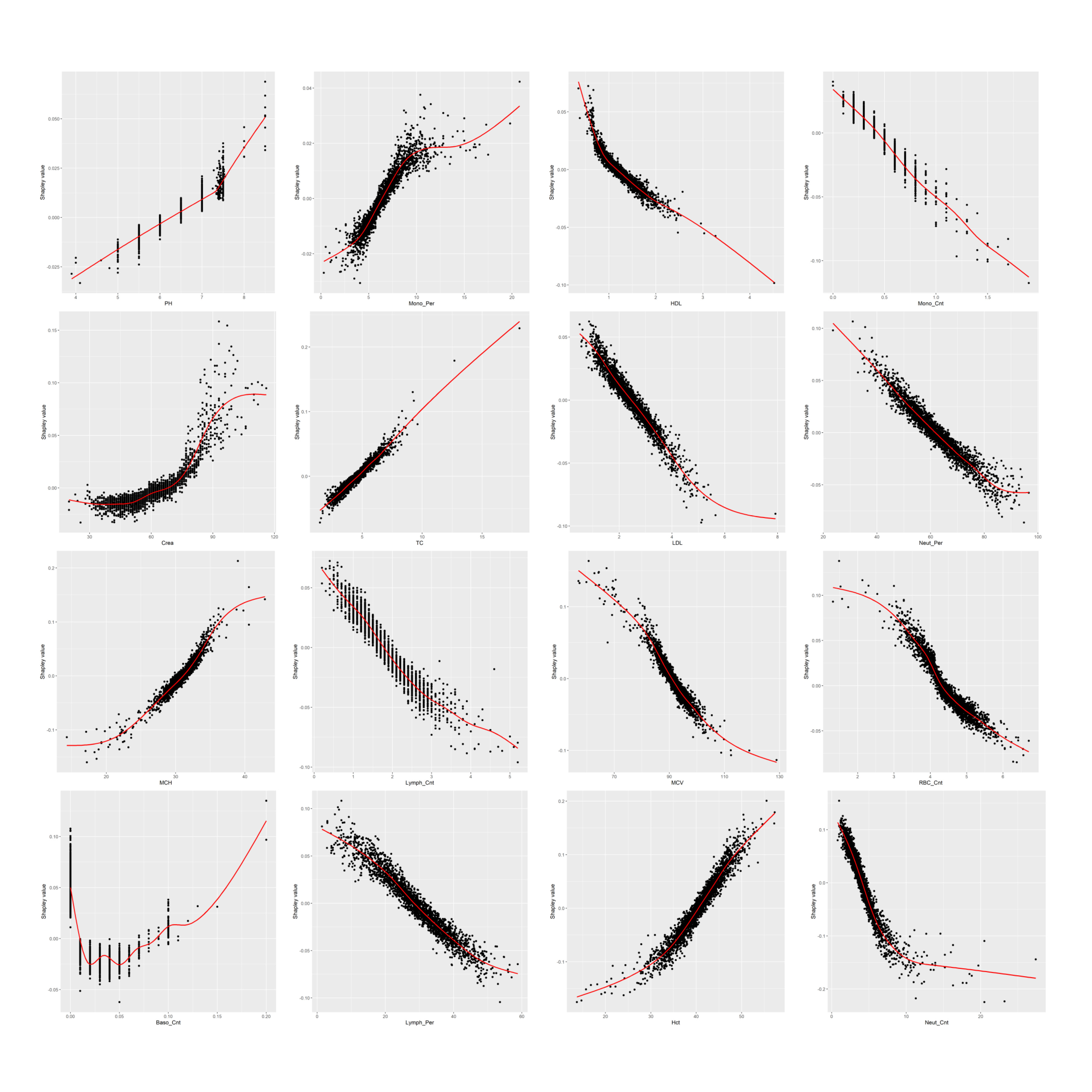

Supplement: Supplementary file 1 — Supplementary Material 1 [file 12911_2025_2977_MOESM1_ESM.jpg]
