# Supplementary material for: A SuperLearner approach for predicting diabetic kidney disease upon the initial diagnosis of T2DM in hospital
Source: BMC Med Inform Decis Mak. 2025 Mar 26;25:148. doi: 10.1186/s12911-025-02977-x (PMC11948915; doi:10.1186/s12911-025-02977-x)
Supplement: Supplementary file 2 — Supplementary Material 2 [file 12911_2025_2977_MOESM2_ESM.docx]

Supplemental materials

**Table S1**. A complete list of the variables in the analysis

| NO | Variables | Abbreviation in analysis (and manuscript) | Unit | Category | Specimen | Variable type | Notes |
| --- | --- | --- | --- | --- | --- | --- | --- |
| 1 | White blood cell count | WBC_Cnt | 10^9/L | Blood routine | whole blood | cont. |  |
| 2 | Neutrophil Counts | Neut_Cnt | 10^9/L | Blood routine | whole blood | cont. |  |
| 3 | Hematocrit | Hct | % | Blood routine | whole blood | cont. |  |
| 4 | Hemoglobin | Hb | g/L | Blood routine | whole blood | cont. |  |
| 5 | Lymphocyte percentage | Lymph_Per | % | Blood routine | whole blood | cont. |  |
| 6 | Basophil counts | Baso_Cnt | % | Blood routine | whole blood | cont. |  |
| 7 | Red blood cell count | RBC_Cnt | 10^12/L | Blood routine | whole blood | cont. |  |
| 8 | Mean corpuscular volume | MCV | fl | Blood routine | whole blood | cont. |  |
| 9 | Lymphocyte count | Lymph_Cnt | 10^9/L | Blood routine | whole blood | cont. |  |
| 10 | Mean Corpuscular Hemoglobin | MCH | pg | Blood routine | whole blood | cont. |  |
| 11 | Neutrophil percentage | Neut_Per | % | Blood routine | whole blood | cont. |  |
| 12 | Low density lipoprotein | LDL | mmol/L | Biochemical analysis | Blood-serum | cont. |  |
| 13 | Age | age | years |  |  | cont. |  |
| 14 | Total cholesterol | TC | mmol/L | Biochemical analysis | Blood-serum | cont. |  |
| 15 | Creatinine | Crea | μmol/L | Biochemical analysis | Blood-serum | cont. |  |
| 16 | Haemoglobin A1c | HbA1c | % | Biochemical analysis | whole blood | cont. |  |
| 17 | Monocyte count | Mono_Cnt | 10^9/L | Blood routine | whole blood | cont. |  |
| 18 | High density lipoprotein | HDL | mmol/L | Biochemical analysis | Blood-serum | cont. |  |
| 19 | Monocyte percentage | Mono_Per | % | Blood routine | whole blood | cont. |  |
| 20 | Urine potentialofhydrogen | PH | - | Urinalysis | Urine | cont. |  |
| 21 | Platelet Distribution Width | PDW | % | Blood routine | whole blood | cont. |  |
| 22 | Eosinophil percentage | Eos_Per | % | Blood routine | whole blood | cont. |  |
| 23 | Total bilirubin | TBIL | μmol/L | Biochemical analysis | Blood-serum | cont. |  |
| 24 | Plateletcrit | PLTHct | % | Blood routine | whole blood | cont. |  |
| 25 | Prealbumin | PA | mg/L | Biochemical analysis | Blood-serum | cont. |  |
| 26 | Triglyceride | TG | mmol/L | Biochemical analysis | Blood-serum | cont. |  |
| 27 | Smoking | is_smoking | - |  |  | bin | 1: yes; 0: no |
| 28 | Mean corpuscular hemoglobin concentration | MCHC | g/L | Blood routine | whole blood | cont. |  |
| 29 | Alanine transaminase | ALT | U/L | Biochemical analysis | Blood-serum | cont. |  |
| 30 | Platelet count | PLT_Cnt | 10^9/L | Blood routine | whole blood | cont. |  |
| 31 | Aspartate aminotransferase | AST | U/L | Biochemical analysis | Blood-serum | cont. |  |
| 32 | D-Dimer | DDimer | ng/ml | Blood coagulation function | plasma | cont. |  |
| 33 | Glucose qualitative | glu_qual | - | Urinalysis | Urine | bin |  |
| 34 | Mean platelet volume | MPV | fl | Blood routine | whole blood | cont. |  |
| 35 | Basophil percentage | Baso_Per | % | Blood routine | whole blood | cont. |  |
| 36 | Red cell volume distribution width -coefficient of variation | RDW-CV  (RDWCV) | % | Blood routine | whole blood | cont. |  |
| 37 | Drinking | is_drinking | - |  |  | bin | 1: yes; 0: no |
| 38 | Ketone qualitative | ket_qual | - | Urinalysis | Urine | bin |  |
| 39 | Aspartate aminotransferase/Alanine transaminase | AST/ALT  (ASTALT) | - | Biochemical analysis | Blood-serum | cont. |  |
| 40 | Calcium | Ca | mmol/L | Biochemical analysis | Blood-serum | cont. |  |
| 41 | Eosinophil count | Eos_Cnt | 10^9/L | Blood routine | whole blood | cont. |  |
| 42 | Urobilinogen qualitative | uro_qual | - | Urinalysis | Urine | bin |  |
| 43 | Nitrite qualitative | nit_qual | - | Urinalysis | Urine | bin |  |
| 44 | Gender | gender | - |  |  | bin | 1: men; 0: women |
| 45 | Occult blood qualitative | ob_qual | - | Urinalysis | Urine | bin |  |
| 46 | [Bilirubin](https://baike.sogou.com/lemma/ShowInnerLink.htm?lemmaId=617381&ss_c=ssc.citiao.link) qualitative | bil_qual | - | Urinalysis | Urine | bin |  |

**Table S2**. Demographic, clinical, and biological characteristics of patients in the training and validation cohorts.

| **Characteristics** | **Training cohort**  **(N = 2303)** | **Validation cohort**  **(N = 988)** | **p-value** |
| --- | --- | --- | --- |
| **Demographics**  **(N = 2)** |  |  |  |
| Age | 61 | 60 | 0.592 |
| Gender |  |  | 0.096 |
| Female | 1240 | 500 |  |
| Male | 1063 | 488 |  |
| **Lifestyle**  **(N = 2)** |  |  |  |
| is_drinking | 286 (12.42%) | 136 (13.77%) | 0.316 |
| is_smoking | 429 (18.63%) | 195 (19.74%) | 0.487 |
| **Blood routine**  **(N = 21)** |  |  |  |
| WBC_Cnt | 6.3 (5.1 - 7.7) | 6.2 (5.175 - 7.7) | 0.624 |
| Neut_Cnt | 3.8 (2.9 - 5) | 3.8 (2.9 - 5.025) | 0.867 |
| Hct | 40.4 (37.3 - 43.7) | 40.3 (37.2 - 43.8) | 0.987 |
| Hb | 137 (126 - 149) | 137 (125 - 149) | 0.998 |
| Lymph_Per | 29 (22.5 - 35.3) | 28.8 (22 - 35.3) | 0.343 |
| Baso_Cnt | 0.02 (0.01 - 0.03) | 0.02 (0.01 - 0.03) | 0.690 |
| RBC_Cnt | 4.42 (4.05 - 4.83) | 4.42 (4.07 - 4.82) | 0.569 |
| MCV | 91.4 (88.6 - 94.2) | 91.2 (88.475 - 93.8) | 0.428 |
| Lymph_Cnt | 1.7 (1.4 - 2.2) | 1.7 (1.3 - 2.2) | 0.152 |
| MCH | 31 (30 - 32) | 31 (29.9 - 32) | 0.532 |
| Neut_Per | 61.9 (54.8 - 68.7) | 62 (55 - 69.6) | 0.344 |
| Mono_Cnt | 0.4 (0.3 - 0.5) | 0.4 (0.3 - 0.5) | 0.725 |
| Mono_Per | 6.3 (5.1 - 7.6) | 6.2 (5.2 - 7.5) | 0.775 |
| PDW | 16.4 (16.1 - 16.7) | 16.4 (16.1 - 16.7) | 0.312 |
| Eos_Per | 1.7 (0.9 - 2.8) | 1.6 (0.9 - 2.6) | 0.248 |
| PLTHct | 0.19 (0.16 - 0.23) | 0.19 (0.16 - 0.23) | 0.571 |
| MCHC | 340 (334 - 345) | 340 (333.075 - 345.225) | 0.906 |
| PLT_Cnt | 197 (162 - 236) | 198 (161 - 239.85) | 0.882 |
| MPV | 9.8 (9 - 10.7) | 9.8 (9 - 10.7) | 0.710 |
| Baso_Per | 0.4 (0.3 - 0.6) | 0.4 (0.3 - 0.6) | 0.775 |
| RDWCV | 12.9 (12.5 - 13.4) | 12.9 (12.4 - 13.4) | 0.566 |
| **Biochemical analysis**  **(N = 13)** |  |  |  |
| LDL | 2.42 (1.87 - 2.955) | 2.43 (1.89 - 2.99) | 0.364 |
| TC | 4.53 (3.835 - 5.24) | 4.54 (3.82 - 5.2925) | 0.547 |
| Crea | 60 (50 - 69) | 59 (49 - 70) | 0.579 |
| HbA1c | 7.4 (6.5 - 9.1) | 7.5 (6.5 - 9.1) | 0.752 |
| HDL | 1.18 (0.97 - 1.42) | 1.17 (0.97 - 1.4) | 0.371 |
| TBIL | 11 (8.2 - 14.7) | 11 (8.1 - 15) | 0.973 |
| PA | 241.5 (196.55 - 289.85) | 242.4 (194.475 - 291.75) | 0.811 |
| TG | 1.41 (1.02 - 2.07) | 1.495 (1.04 - 2.1825) | 0.066 |
| ALT | 20 (14 - 32) | 21 (14 - 32) | 0.425 |
| AST | 20 (16 - 26) | 20 (15 - 28) | 0.674 |
| ASTALT | 0.9 (0.7 - 1.3) | 0.9 (0.7 - 1.25) | 0.893 |
| Ca | 2.25 (2.16 - 2.34) | 2.25 (2.16 - 2.34) | 0.889 |
| Eos_Cnt | 0.1 (0.06 - 0.18) | 0.1 (0.05 - 0.17) | 0.190 |
| **Urinalysis**  **(N = 7)** |  |  |  |
| pH | 6 (5.5 - 6.5) | 6 (5.5 - 6.5) | 0.604 |
| glu_qual | 878 (38.12%) | 384 (38.87%) | 0.717 |
| ket_qual | 242 (10.51%) | 104 (10.53%) | 1 |
| uro_qual | 22 (0.96%) | 7 (0.71%) | 0.624 |
| nit_qual | 44 (1.91%) | 19 (1.92%) | 1 |
| ob_qual | 256 (11.12%) | 100 (10.12%) | 0.435 |
| bil_qual | 9 (0.39%) | 2 (0.2%) | 0.597 |
| **Blood coagulation function (N = 1)** |  |  |  |
| DDimer | 109 (63 - 207.5) | 116 (68 - 223.25) | 0.0565 |

Footnotes: Continuous variables are presented as medians (interquartile ranges), and categorical variables are presented as counts (percentages).

**Table S3**. AUC performance comparison between SuperLearner and other models

| **NO** | **SuperLearner AUC** | **Other AUCs** | **p-value*** |
| --- | --- | --- | --- |
| 1 | 0.7138 [0.673, 0.7546] | MLR: 0.6682 [0.6251, 0.7113] | 0.001915 |
| 2 | 0.7138 [0.673, 0.7546] | Lasso 0.6684 [0.6237, 0.7131] | 0.005048 |
| 3 | 0.7138 [0.673, 0.7546] | Random forest 0.682 [0.6397, 0.7258] | 0.00876 |
| 4 | 0.7138 [0.673, 0.7546] | XGBoost 0.6783 [0.6376, 0.72] | 0.003038 |

*roc.test() for two Receiver Operator Characteristic (ROC) curves

**Fig S1.** Shapley additive explanation dependence plot of SuperLearner (the remaining 16 from the top 20), depicting how a single variable affects the prediction.

(Footnotes: The abbreviations of all analytical variables are detailed in **Table S1**)
